# Effects of High-Pressure Homogenization on the Structural, Physical, and Rheological Properties of Lily Pulp

**DOI:** 10.3390/foods8100472

**Published:** 2019-10-10

**Authors:** Jie Liu, Rongrong Wang, Xinyu Wang, Lvzhu Yang, Yang Shan, Qun Zhang, Shenghua Ding

**Affiliations:** 1 Longping Branch Graduate School, Hunan University, Changsha 410125, Chinawxy25994@163.com (X.W.);; 2 Hunan Agricultural Product Processing Institute, Hunan Academy of Agricultural Sciences, Hunan Provincial Key Laboratory for Fruits and Vegetables Storage Processing and Quality Safety, Changsha 410125, China; sy6302@sohu.com (Y.S.); zqun208@163.com (Q.Z.); 3 College of Food Science and Technology, Hunan Agricultural University, Changsha 410128, China; sdauwrr@163.com

**Keywords:** high-pressure homogenization, lily pulp, physical properties, rheological properties

## Abstract

The effects of high-pressure homogenization (HPH) on the structural, physical, and rheological properties of lily pulp (15%, *w*/*w*) were investigated. Different pressures ranging from 0 MPa to 100 MPa were used. The focus was on evaluating the changes in the particle size distribution (PSD), structure, pulp sedimentation behavior, serum cloudiness (SC), total soluble solids (TSS), color, and rheological behavior of the pulps. PSD analysis showed that the diameter of suspended lily particles significantly decreased with an increasing homogenization pressure. The suspended particles observed through optical microscopy became small after homogenization, highlighting the effect of HPH on disrupting the suspended particles. Compared with the untreated pulp, the SC and sedimentation velocity of the homogenized pulps decreased due to the disruption of the suspended particles. The effects of HPH on the sedimentation index and SC exhibited an asymptotic behavior similar to that of the changes in the particle size of lily pulp. Moreover, HPH processing reduced the viscosity of lily pulp and increased the TSS and lightness of the homogenized pulps. HPH significantly modified the structural, physical, and rheological properties of lily pulp. The pulp homogenized above 60 MPa had good suspension stability. This finding indicates that HPH technology can be used to improve the stability of lily pulp.

## 1. Introduction

Lily (*Lilium brownii var. viridulum*), a perennial ornamental crop that originated from Northern and Eastern Asia, Europe and North America in the Northern Hemisphere, and China, is known as the diversity center of the genus *Lilium* with about 55 species worldwide [1]. In addition to its economic importance and popularity in horticulture, lily bulbs contain abundant starch, protein, glucomannan, and various bioactive compounds, such as polysaccharides, phenolics, saponin, and terpenoids [2,3], which exhibit multiple health-promoting properties, including antioxidant, anti-inflammatory, and immunity-enhancing effects [4,5]. Thus, lily is commonly consumed as an important edible and medicinal plant. Lily bulbs have high nutritional and pharmacological values and are rich in dietary fibers and low in fat; hence, they are important plant resources for developing novel nutritionally improved products, such as lily-based vegetable beverages [6,7]. Nowadays, the demand for vegetable-based beverage products is increasing due to changes in people’s food preferences and the increasing number of problems related to the intolerance for cow’s milk [8].

High-pressure homogenization (HPH) is considered one of the most promising technologies for manufacturing beverages due to improvements in HPH and increasing consumer acceptance of pressure-processed food [9]. HPH is a non-thermal technology wherein a fluid is forced through a narrow gap under conditions of high pressure; its velocity is considerably increased, resulting in depressurization with consequent cavitation and high shear stress [10]. HPH is widely used in food, cosmetic, and pharmaceutical industries primarily to emulsify, mix, and disperse substances [11]. In particular, the HPH technique is an appropriate means of producing various liquid products, such as cream, fruit juices, and milk or milk-based and vegetable-based beverages because it promotes physical stability and limits thermal damage [12]. In previous studies, HPH was used to modify several physical properties of fluids and introduce novel changes, including reducing particle size and phase separation, changing the rheological behavior, and improving texture uniformity [13,14]. Notably, the rheological properties of fluid food are important factors that affect development and optimization processes and determine the product’s appearance, taste, texture, and shelf-life; consequently, they also influence the product’s sensory quality and consumer acceptance [15]. Several studies have investigated HPH-induced changes in the particle size distribution (PSD) and rheological behavior of numerous fruits or vegetable products, such as almonds and hazelnut milk [8], tomato juices [16], cashew apple juices [17], carrot juices [18], orange pulp [19], and parsnip suspensions [20]. These previous studies suggested that HPH processing causes changes in the particle size, particle shape, and PSD of samples and consequently influences the overall physical properties and rheological behavior of samples.

However, although the effects of HPH on physical and rheological property changes in numerous milk drinks, fruit juices, and vegetable beverages have been widely investigated, only a few studies have focused on physical changes in samples mixed with proteins and starches after HPH processing. Lily pulp is a composite system that consists of starches, proteins, and dietary fibers; these dispersed particles as quality parameters are significantly affected by various factors in process control, such as PSD, microstructure, particle surface charge, and total soluble solids (TSS) [21]. Yu et al. investigated the effects of HPH on the physical and rheological properties of taro pulp (consisting mainly of carbohydrates and proteins) [9,22]. The results showed that the changes in macromolecular structure and the interaction of taro proteins and starches caused by HPH processing directly influence the rheological behavior of pulp. The findings suggested that HPH can be used to improve the suspension stability of taro and lily pulps. Other studies have reported that starch–protein or starchy products subjected to HPH processing cause starch gelatinization and protein denaturation, which lead to strong gel networks and novel rheological properties of products [23,24,25].

The objective of the present work was to investigate the influence of HPH treatment on the PSD, microstructure, pulp sedimentation, serum cloudiness (SC), color, TSS, and rheological characteristics of lily pulp. The study also evaluates the potential use of HPH in the industrialized manufacturing of lily-based beverages and defines the processing conditions that would ensure product quality and stability.

## 2. Materials and Methods

### 2.1. Materials and Sample Preparation

Fresh lily bulbs (*Lilium lancifolium* Thunb.) were collected from Longshan County, Hunan Province, China (29°38′ N, 109°25′ E). The lily bulbs were checked for insects, pests, diseases, and mechanical injury before being washed, drained, and sliced. A blanching treatment was conducted in boiled water for 150 s to inactivate browning-related enzymes in accordance with the pre-experiments. The lily pulp (15%, *w*/*w*) was prepared by mixing the blanched lily slices with sterile distilled water and grinding the mixture for 1 min in a domestic blender (JYL-Y5, Joyoung, Shandong, China). The obtained lily pulp was made to pass through a 60-mesh sieve to remove impurities and coarse particles and stored at 4 °C until used.

### 2.2. Homogenization of Lily Pulp

Exactly 500 mL of lily pulp was homogenized for one pass at different pressures of 0, 20, 40, 60, 80, and 100 MPa by using a laboratory-scale high-pressure homogenizer (Nathox Lab 3, Nathan Technologies, Pittsburgh, PA, USA) in triplicate. After HPH, the obtained samples were added with citric acid (0.2%, *w*/*w*) and potassium sorbate (0.4%, *w*/*w*) to avoid microbial growth during the 30 d of storage evaluation at 25 °C.

### 2.3. Particle Size Analysis

The PSDs of the non-homogenized (NH) and homogenized samples (0–100 MPa) were determined via laser diffraction (LS13320, Beckman Coulter Instrument, Miami, FL, USA). Before the measurements, the lily pulp was diluted in ultrapure water in a diffractometer cell from an aqueous liquid module (ALM) until the obscuration rate reached 40%. The mean particle and cumulative diameters (Dv10, Dv90, and Dv50, indicating that 10%, 50% or 90% of the particles fell below the specified diameter) were obtained [26].

### 2.4. Microstructure of Homogenized Lily Pulp

The homogenized samples (0–100 MPa) and NH were observed under 50× magnification by using an optical microscope (Moticam Pro 205A, Motic China Group, Shenzhen, China) equipped with a charge-coupled device camera module to evaluate the effects of HPH on the microstructure of the lily pulp. The samples were carefully placed on glass slides and covered with a cover slip. Then, images of each sample were captured at least in triplicate.

### 2.5. Pulp Sedimentation

After HPH treatment, three samples of NH and homogenized (0–100 MPa) pulp were transferred to 25 mL graduated tubes (sterilized), and a pulp sedimentation test was conducted at a controlled temperature of 25 °C for 30 d. The sedimentation index (IS) was obtained with Equation (1), as described by Kubo et al. [10] and Silva et al. [27].

(1) IS=sedimentation volume/total sample volume

### 2.6. Serum Cloudiness

For each sample of lily pulp, SC was measured after centrifuging at 10,000× *g* for 10 min at 20 °C (Avanti J-26 XP, Beckman Coulter, Boulevard Brea, CA, USA). The optical density of the supernatant was read using a spectrophotometer (UV-1800, Shimadzu, Suzhou, China) at 660 nm with distilled water as the reference. The absorbance reading was directly related to SC [27,28].

### 2.7. Rheological Behavior

Rheological determinations were carried out with a rotational rheometer (MCR302, Anton Paar, Graz, Steiermark, Austria) equipped with concentric cylinders in accordance with the method of Calligaris et al. but with some modification [29]. The temperature was constantly controlled at 25 °C by using a Peltier system. The samples were subjected to a steady-flow test at shear rates ranging from 0 s^−1^ to 300 s^−1^, and pulp flow behavior was fitted to the Herschel–Bulkley model (Equation (2)) as follows:

(2) $$\sigma =\sigma_{0}+k{\left(\gamma \right)}^{n}$$

where *σ* is the shear stress (Pa), *σ*_0_ is the yield stress, *γ* is the shear rate (s^−1^), *k* is the consistency index (Pa.s*^n^*), and *n* is the flow behavior index (dimensionless) [30].

### 2.8. Determination of Color and Total Soluble Solids

The instrumental color of the lily pulp was determined using a Color Quest XE colorimeter (ColorQuest XE, Hunter Associates Laboratory, Reston, VA, USA). After calibrating the instrument with a standard black-and-white tile, the glass cell containing the samples was placed above the light source for measurement. Then, the CIE (Coherent Infrared Energy) values of L* (lightness), a* (redness and greenness), and b* (yellowness and blueness) were obtained [31], and the total color difference (*ΔE*) between the NH and homogenized samples was calculated with Equation (3) [9], where *L*_0_*, *a*_0_*, and *b*_0_* are the values of the NH samples.

(3) $$∆E=\sqrt{{\left(L^{*}-L_{0}^{*}\right)}^{2}-{\left(a^{*}-a_{0}^{*}\right)}^{2}-{\left(b^{*}-b_{0}^{*}\right)}^{2}}$$

The TSS of lily pulp was measured as °Brix with a digital refractometer (WZB 45, Shanghai INESA Physical-Optical Instrument Co., Shanghai, China) at room temperature.

### 2.9. Statistical Analysis

All experiments were performed in triplicate, and the data were subjected to one-way analysis of variance (ANOVA) using SPSS 20.0. The results were expressed as mean ± standard deviation (SD), and the statistical significance was set to *p* < 0.05.

## 3. Results and Discussion

### 3.1. Changes in the Particle Size Distribution of Lily Pulp Treated with HPH

**Figure 1:**
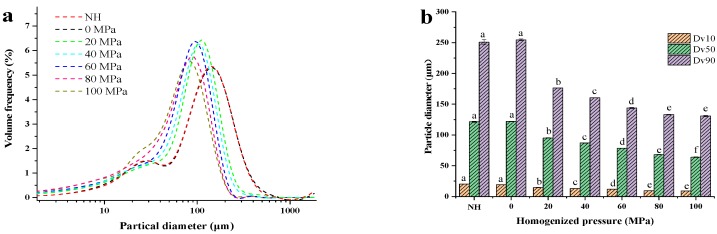
Effect of high-pressure homogenization (NH = non-homogenized; 0–100 MPa) on particle size distribution (PSD) (**a**) and cumulative diameter percentiles (Dv10, Dv50, and Dv90) (**b**) of lily pulp. Dv10, Dv50, and Dv90 indicated that 10%, 50% or 90% of the particles fell below the specified diameter. Different letters in the column indicated that the samples were significantly different (*p* < 0.05).

Figure 1 presents the effect of HPH on the PSD of lily pulp. Figure 1a indicates that the PSD behavior of the NH sample exhibited a bimodal distribution phenomenon, and the particle diameters of the two main peaks were 33.01 and 133.74 μm. A similar phenomenon has been observed in tomato paste suspensions [32]. Furthermore, the sample homogenized at 0 MPa exhibited the same PSD behavior as the NH sample. This result indicates that the lily pulp passing through the homogenizer without pressure caused no particle disruption, which is in agreement with previous observations for taro pulp [9] and tomato juice [10]. Compared with the control (NH and homogenized at 0 MPa) samples, the PSD shape of the samples homogenized at 20–100 MPa had a narrower distribution with increasing pressure, which indicated more uniformity. As expected, the mean particle size decreased with the increase in pressure during homogenization (up to 80 MPa), and the mean particle diameter decreased from 132.78 μm to 68.40 μm. Similar results have been obtained in vegetable products, such as tomato-based ones (up to 9 MPa; up to 60 MPa) [33,34] and carrot and broccoli dispersions (up to 60 MPa) [35]; numerous fruit products, such as apple (up to 300 MPa) [36], orange (up to 150 MPa) [37], and citrus (up to 30 MPa) juices [38]; and milk products, such as bovine milk (up to 300 MPa) [39] and acidified milk beverages (up to 30 MPa) [40]. Figure 1b reflects the changes of the cumulative diameter percentiles (Dv10, Dv50, and Dv90) with increasing pressure, which shows that HPH processing progressively decreased the values of Dv10, Dv50, and Dv90; similar results have been obtained in previous studies on tomato puree after HPH processing [41]. The changes in PSD between 0 and 60 MPa were more pronounced than those between 60 and 100 MPa. In other words, PSD showed small changes with the increase in homogenization pressure at high pressures. Thus, the effect of HPH on the disruption of particles appeared to demonstrate an asymptotic behavior.

### 3.2. Changes in the Microstructure of Lily Pulp Treated with HPH

**Figure 2:**
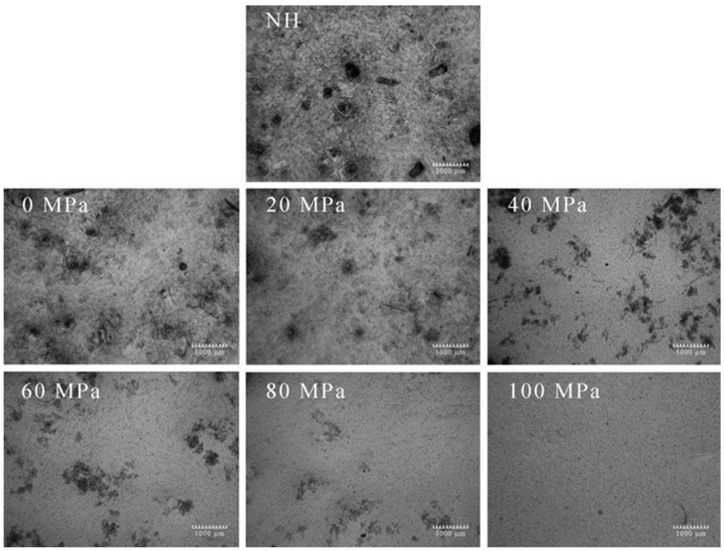
Effect of pressure of high-pressure homogenization on the microstructure of lily pulp. The scale bar shows 1000 μm.

Figure 2 presents the changes in microstructure between the NH and homogenized samples obtained by optical microscopy. The sample homogenized at 0 MPa had a similar structure as the NH sample, and both of them contained large amounts of deformable and small particles. The large particles were associated with several completed cells or aggregates, and the small ones were related to starch granules, fibrous particles, cell walls, or internal constituents [27]. An obvious difference was observed between the control (NH and homogenized at 0 MPa) samples and the HPH-treated (20–100 MPa) ones. The large tissue fragments were progressively reduced to small pieces with the increase in pressure during HPH treatment. In addition, Figure 2 (60, 80, and 100 MPa) indicates that the lily pulps allowed for complete cell disruption while the pulps were homogenized above 60 MPa.

As expected and confirmed by the PSD analysis, the suspended particles were broken down into the small particles at a high homogenization pressure, causing the lily pulp to have many small particles, such as polymers, fiber fractions, cellular material, and cell wall fragments [9]. This finding emphasizes the effect of HPH on disrupting vegetable pulp particles. Tan and Kerr reported similar results in tomato puree; they found that HPH processing produces increasingly small and highly uniform suspended particles with increasing pressure and pass number [41]. The changes in microstructure induced by HPH processing have been investigated not only in various fruit or vegetable products, but also in numerous protein beverages and products, such as soymilk mixture [42] and non-fat and low-fat yoghurts [43]. These previous studies have illustrated that homogenization processing induces several structural changes in particles dispersed in colloidal suspensions, which in turn influence the overall physical properties.

### 3.3. Changes in the Pulp Sedimentation of Lily Pulp Treated with HPH

**Figure 3:**
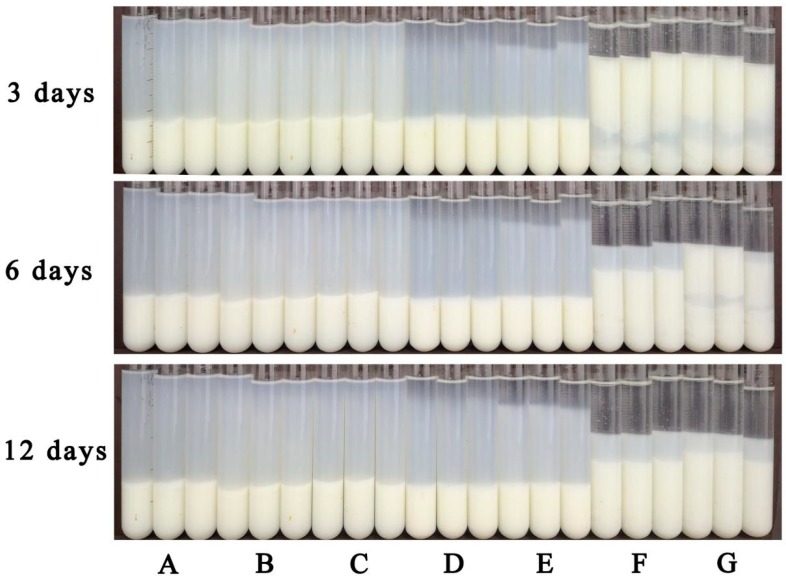
Macroscopic observations of NH (non-homogenized) sample and high-pressure homogenization (0–100 MPa) processed samples after 3, 6, and 12 d at 25 °C: (**A**) 100 MPa; (**B**) 80 MPa; (**C**) 60 MPa; (**D**) 40 MPa; (**E**) 20 MPa; (**F**) 0 MPa; (**G**) NH, and each capital letter (A to G) refers to a triplicate of tubes.

Figure 3 shows the macroscopic appearance of the NH sample and samples treated by homogenization at different pressures and stored for 3, 6, and 12 d at 25 °C. After the first day of storage, only the NH sample and the samples homogenized at 0 MPa showed phase separation, which is commonly observed in several fruit or vegetable pulps, such as pineapple pulp [27]. The sedimentation velocity of dispersed particles was progressively reduced when the homogenization pressures reached 60 MPa during HPH processing, similar to previous observations of tomato juice [10] and cashew apple juice [17]. This result indicates that HPH is a valuable tool to prevent pulp sedimentation and improve the stability of lily pulp.

**Figure 4:**
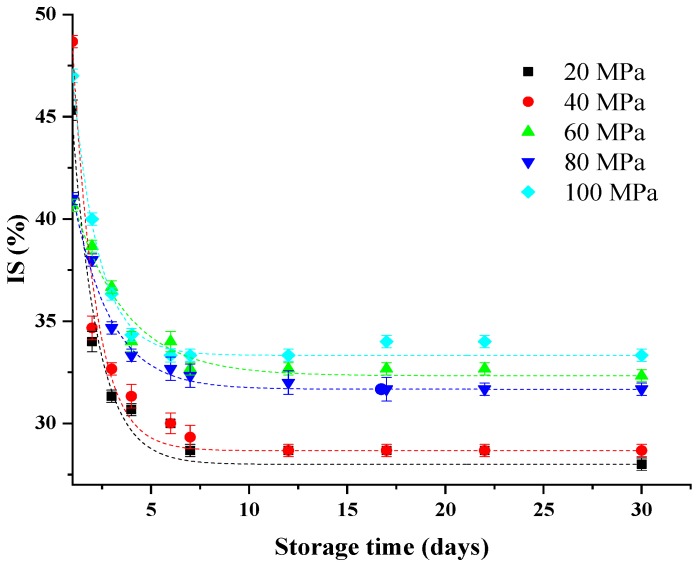
Effect of high-pressure homogenization on the sedimentation index (IS) of lily pulp (20–100 MPa) during 30 days of storage at 25 °C: the dashed curves are the models described in Table 1.

The effect of HPH on the IS of samples (20–100 MPa) with storage time is shown in Figure 4. As expected, the rate of sedimentation as a function of storage time demonstrated asymptotic behavior, as evidenced by the results on the mean particle diameter at different homogenized pressures in Figure 1. The IS of the pulps was subsequently modeled as a function of storage time by using the exponential decay function, which had a high coefficient of determination (*R*^2^ > 0.95). The related model and parameters are displayed in Table 1. The main changes in IS occurred in the first seven days. Moreover, the pulp homogenized at 100 MPa demonstrated quicker sedimentation than the 60 MPa sample during the first six days. The pulp homogenized at 80 MPa had a lower IS than the 60 MPa sample throughout the 30 days. These results were obtained because the low viscosities of the pulps were insufficient to maintain the stability of the suspensions. Stokes’ law states that the settling velocity of spherical particles depends on the functional properties of the particles and suspending medium. According to this law, the sedimentation velocity of a dispersed particle is proportional to its diameter and the difference between the densities of particles and the continuous medium; meanwhile, it is inversely proportional to the viscosities of the continuous medium [17]. Notably, surface forces, such as Van der Waals and electrostatic forces, are crucial for small particles. Therefore, small particles obtained at high homogenization pressures are expected to form large aggregates due to the forces of attraction, even if the sizes of particles are not in the colloidal domain [27]. In conclusion, the smaller particles are, the stronger the attractive surface forces are, which would result in larger aggregates and faster pulp sedimentation at the higher homogenized pressures. A similar behavior was observed in a previous study on pineapple pulp; the sedimentation tests showed the highest stability for samples homogenized between 200 and 300 bar, but the pulp had higher sedimentation velocity and phase separation above 400 bar [27].

### 3.4. Changes in Serum Cloudiness of Lily Pulp Treated with HPH

**Figure 5:**
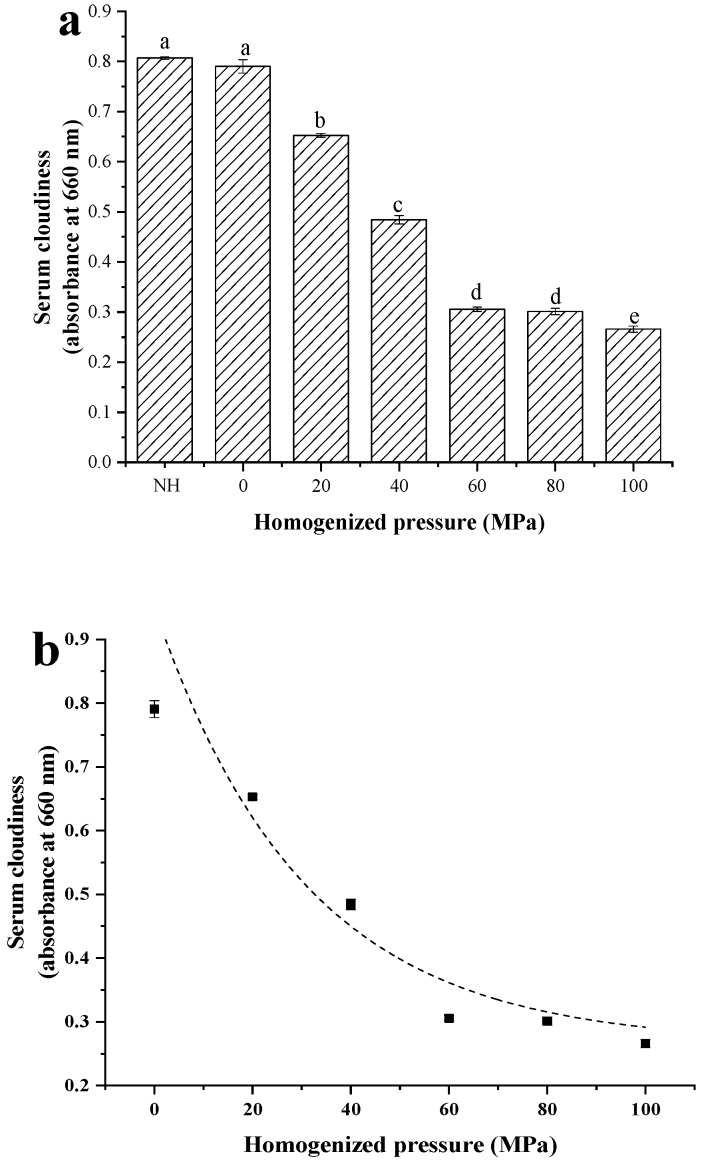
Effect of high-pressure homogenization on serum cloudiness (SC) of lily pulp (**a**) and the dashed curve is SC of the pulps was modeled as a function of homogenization pressure (**b**). Different letters in the column indicated that the samples were significantly different (*p* < 0.05).

The changes in the SC of samples caused by homogenization processing in lily pulp are shown in Figure 5a. The cloudiness decreased when the pressure increased because of the disruption of the dispersed particles during HPH treatment. Given that the smaller dispersed particles allowed more light to pass through the pulp serum, leading to lower absorbance values, the decrease in SC appeared to be due to the reduction in particle size, which is in accordance with the previous observation for PSD behavior. Figure 5b illustrates the relationship between homogenization pressure (0–100 MPa) and SC. The effect of homogenization pressure on the lily pulp’s SC also demonstrated asymptotic behavior. This observation is in agreement with those of previous studies on taro pulp [9], tomato juice [10], and pineapple pulp [27]. The SC of the pulps was modeled as a function of homogenization pressure by using the exponential decay function with a high coefficient of determination (*R*^2^ > 0.93). The obtained model and parameters are presented in Table 2.

### 3.5. Changes in the Total Soluble Solids and Color of Lily Pulp Treated with HPH

**Figure 6:**
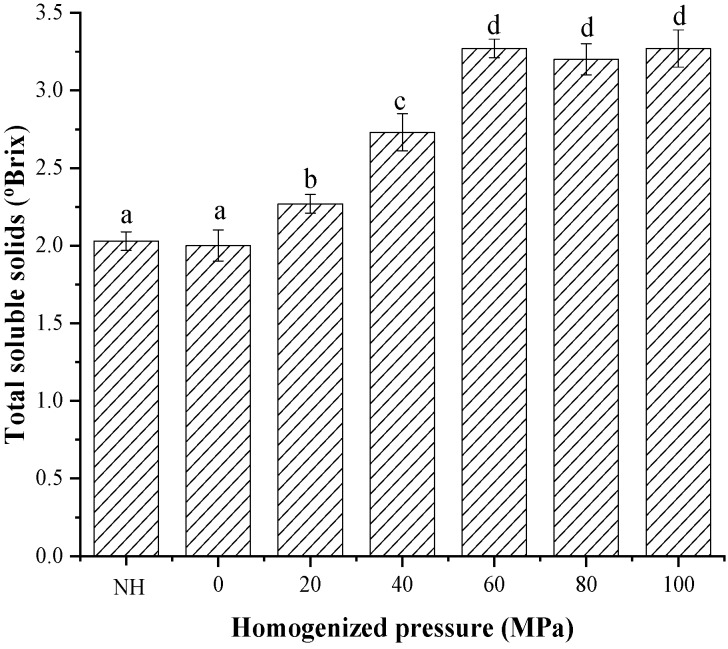
Effect of high-pressure homogenization on the total soluble solids (TSS) content of lily pulp. Different letters in the column indicate that the samples were significantly different (*p* < 0.05).

The effect of HPH treatment on TSS is shown in Figure 6. As expected, HPH processing caused a significant increase in the TSS of the homogenized samples, which increased from 2.00 °Brix to 3.27 °Brix with the homogenization pressure of 60 MPa. Similar results have been obtained in previous studies on homogenized taro pulps [9]. In general, lily pulp is a composite suspended system that contains starch-filled cells, parenchyma cells, or various cell aggregates dispersed in continuous liquid phase; the release of starch granules from starch-filled cells during the boiling and squeezing is greatly limited because of the surrounding cells and consequently limits the extent of lily starch granule swelling and gelatinization [44]. Hence, the increase in the TSS of the homogenized samples could be associated with the effect of mechanical and thermal stress on the pulps during HPH treatment. More starch granules were released, and the extent of lily starch granule swelling and gelatinization was consequently increased. Determination of the microstructure and PSD behavior showed that the suspended lily particles decreased in size with increasing homogenization pressure. This observation is similar to those of previous studies on the changes in the physical properties of wheat starch [45], cassava starch [46], and maize starch [47] after HPH processing.

The parameters of L* (lightness), a* (redness: red to green), and b* (yellowness: yellow to blueness) and the total color differences (*ΔE*) are presented in Table 3. HPH processing induced an increase in L* values and a decrease in b* and a* values, but the a* values between the NH and HPH-treated samples showed no significant differences. These results indicate that the HPH-treated pulps appeared brighter and had less yellowness, but the effect of HPH treatment on the redness of lily pulps was not obvious. Caponio et al. also obtained high L* values after HPH treatment of olive-based pâtés [48]. Given that homogenization disrupts the remaining complete cells and breaks the fragments into small dispersed particles, small pulp particles allow more light to scatter and result in an increase in the lightness of lily pulps. Similar observations have been obtained from homogenized taro pulp [9] and several fruit juices, such as tomato [10], mango [49], and banana [32]. In addition, Cserhalmi et al. [50] reported that the parameter of *ΔE* can reflect the extent of color differences between homogenized and NH samples. The parameter can be divided into five levels, namely, great (6.0 to 12.0), well visible (3.0 to 6.0), noticeable (1.5 to 3.0), slightly noticeable (0.5 to 1.5), and not noticeable (0 to 0.5). In the current case, the lily pulps homogenized at pressures between 40 and 100 MPa presented a noticeable variation in color in comparison with the control sample (NH), and those homogenized at pressures between 0 and 20 MPa presented slightly noticeable differences in color.

### 3.6. Changes in the Rheological Properties of Lily Pulp Treated with HPH

**Figure 7:**
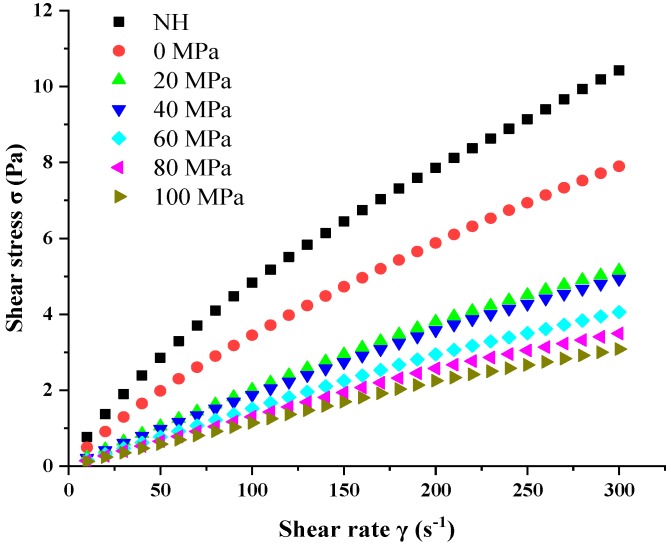
Steady shear flow curves (*σ × γ)* of lily pulp treated by high pressure homogenization (0–100 MPa) and NH (non-homogenized) sample.

The flow curves (*σ × γ*) of the NH and homogenized pulps (0–100 MPa) at 25 °C are shown in Figure 7. HPH processing decreased the shear stress (*σ*) of the pulps in relation to each shear rate (*γ*). As expected, the lily pulps’ flow behavior was well fitted to the Hershel–Bulkley rheological model (*R*^2^ > 0.995), and the parameters obtained for this model are summarized in Table 4. The NH and homogenized pulps exhibited pseudoplastic behavior with the presence of yield stress (*σ_0_*). *σ_0_* is one of the important quality parameters that characterize the properties of semi-solid foods [51]. The *σ_0_* and consistency index (*k*) of the lily pulps significantly decreased, and the flow behavior index (*n*) increased after HPH processing. Several studies have shown similar product *n* and *k* changes in relation to PSD behavior changes. For example, Leite et al. reported that a decrease in particle size caused by HPH promotes a decrease in cashew apple juice consistency, *σ*_0_, and *k* and an increase in *n* [17]. As the homogenization pressure increased from 0 MPa to 100 MPa, the *σ*_0_ value of lily pulp decreased from 0.772 Pa to 0.125 Pa, indicating that the homogenized pulps required low shear stress to initiate the pulp flow. The value of *k* decreased from 0.088 Pa·s*^n^* to 0.012 Pa·s*^n^*, suggesting that the viscosity of lily pulp decreased with increasing homogenization pressure at rest. The value of *n* increased from 0.826 to 0.926, which indicated that the homogenized pulps demonstrated a flow behavior that is close to Newtonian behavior.

**Figure 8:**
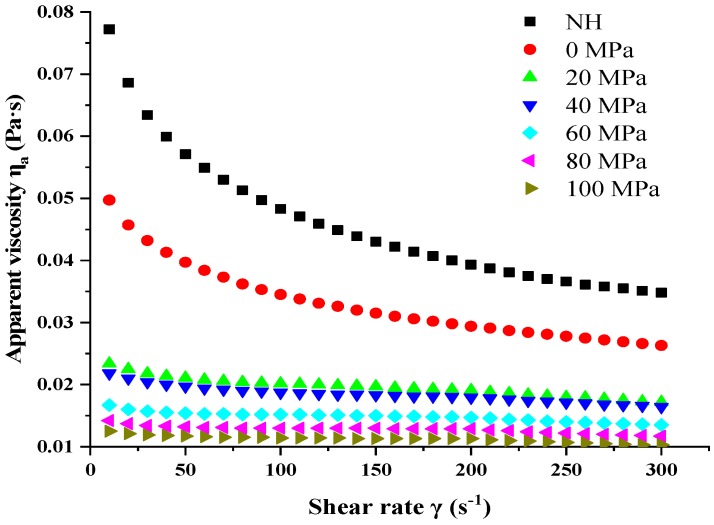
Apparent viscosity (*η_a_ × γ*) of lily pulp treated by high-pressure homogenization (0–100 MPa) and NH (non-homogenized) sample.

The relevant behavior of the apparent viscosity (*η_a_*) of NH and HPH-treated pulps as a function of shear rate (*η_a_ × γ*) is shown in Figure 8. The *η_a_* of all the pulps decreased with increasing shear rate, which suggested that the NH and homogenized samples exhibited shear thinning behavior. Meanwhile, the apparent viscosities of the lily pulps treated by HPH were lower than those of the NH pulp over the entire shear rate range. These observations suggest that HPH processing changed the rheological properties of the lily pulp and led to a decrease in the lily pulp’s *η_a_*. Several studies have also reported decrements in the consistency of samples after HPH processing, such as tomato puree [41], banana juice [29], and orange juice [52].

To the best of our knowledge, lily pulp is a two-phase system that contains the aqueous serum phase and the dispersed phase with a complicated rheology, which indicates that the effect of HPH can occur in each of these phases. In general, the aqueous serum phase contains acids, salts, sugars, and several soluble polysaccharides but is equal to a Newtonian fluid. The dispersed phase contains cell clusters, single cells, fragmented cells or cell remnants, and colloidally suspended polysaccharides [3]. The flow behaviors of samples are mainly influenced by hydrodynamic forces and minimally affected by Brownian motion (a random motion of particles suspended in a fluid resulting from their collision with the quick atoms or molecules in the gas or liquid) and inter-particle forces. Moreover, PSD and shape are crucial factors in this system because the system is best regarded as a non-colloidal dispersion due to the size of particles (mostly above 10 μm). Augusto et al. [53] reported that the viscosity of tomato juice serum decreases as the homogenization pressure increases because of the disruption of molecules. Similar results are expected for the lily pulp serum phase. That is, the changes in the rheological properties of lily pulp are dominated by the changes in particles of the dispersed phase. Hence, determinations of PSD and microstructure showed that the particles dispersed in lily pulp were reduced in size at high homogenization pressures. These observations can also illustrate the decrease in lily pulp viscosity because small particles can occupy the spaces among large ones, leading to an important lubricant effect for reducing the resistance to flow and consequently resulting in a decrease in *k* [54]. However, many previous studies have revealed increments in the viscosity of samples treated by HPH processing, such as taro pulp [22] and mango products [49,55], which suggests that the effect of HPH differs for each material and highlights the importance of investigating this process further.

## 4. Conclusions

In this study, the effect of HPH on the structural, physical, and rheological properties of lily pulp were evaluated. HPH processing altered the PSD, microstructure, SC, pulp sedimentation behavior, and color of the lily pulp. The particle size significantly decreased after HPH processing because the homogenization pressure disrupted the suspended particles and consequently affected other physical and rheological properties. The microstructure images obtained with an optical microscope confirmed the results of the PSD analysis. Determination of SC and pulp sedimentation showed that the absorbance of the serum phase and the sedimentation velocity significantly decreased with an increase in homogenization pressure and exhibited asymptotic behavior. HPH processing increased the TSS and lightness of lily pulp, but the *ΔE* values did not exceed 3.0. Moreover, the disruption of suspended particles modified the rheological characteristics of lily pulp. For example, HPH increased *n* and decreased *σ*_0_ and *k*. These results indicate that HPH processing reduced the consistency of lily pulp and thus caused decreased energy consumption. Therefore, HPH technology could be used as a tool to improve the quality of lily pulp.

## Figures and Tables

**Table 1:** Mathematical modeling of the sedimentation index (IS) of the homogenized lily pulp during 30 days of storage at 25 °C.

Treatments	IS = IS_equilibrium_ + (IS_initial_ − IS_equilibrium_)·e^−k·t^			
	IS_equilibrium_	IS_initial_	k	*R* ^2^
20 MPa	28.00	64.09	0.77	0.95
40 MPa	28.67	75.69	0.86	0.99
60 MPa	32.33	44.91	0.38	0.98
80 MPa	31.67	47.78	0.52	0.99
100 MPa	33.33	62.97	0.78	0.99

IS in % and t in days.

**Table 2:** Mathematical modeling of serum cloudiness (SC) as a function of the homogenized pressure (0–100 MPa) in lily pulp.

Model	SC = SC_equilibrium_ + (SC_initial_ − SC_equilibrium_)·e^−k·p^
SC_equilibrium_	0.27
SC_initial_	0.95
K	0.03
R^2^	0.93

SC in % and p (pressure) in MPa.

**Table 3:** Mean (±SD) values of lightness (L*), redness (a*), yellowness (b*), and total color differences (Δ*E*) of the NH (non-homogenized) sample and high-pressure homogenization (0–100 MPa) processed lily pulps.

Treatments	L*	a*	b*	*ΔE*
NH	67.03 ± 0.19 ^c^	−3.30 ± 0.03 ^ab^	2.48 ± 0.10 ^a^	-
0 MPa	67.78 ± 0.26 ^b^	−3.31 ± 0.03 ^ab^	2.02 ± 0.03 ^b^	0.88 ± 0.08 ^d^
20 MPa	67.81 ± 0.28 ^b^	−3.32 ± 0.04 ^bc^	1.99 ± 0.03 ^b^	0.92 ± 0.10 ^d^
40 MPa	68.80 ± 0.27 ^a^	−3.32 ± 0.02 ^bc^	1.53 ± 0.01 ^c^	2.01 ± 0.09 ^b^
60 MPa	69.11 ± 0.16 ^a^	−3.26 ± 0.02 ^a^	1.40 ± 0.04 ^de^	2.34 ± 0.05 ^a^
80 MPa	68.60 ± 0.22 ^a^	−3.38 ± 0.03 ^d^	1.51 ± 0.02 ^cd^	1.85 ± 0.06 ^c^
100 MPa	68.67 ± 0.06 ^a^	−3.37 ± 0.02 ^cd^	1.44 ± 0.05 ^d^	1.94 ± 0.01 ^bc^

Different letters in the same column indicate significant difference (*p* < 0.05) between treatments.

**Table 4:** Herschel–Bulkley model parameters of the control and homogenized lily pulps (0–100 MPa, 25 °C).

Treatments	*σ*_0_ (Pa)	*k* (Pa.s^*n*^)	*n*	*R* ^2^
NH	0.772 ± 0.030 ^a^	0.088 ± 0.007 ^a^	0.826 ± 0.015 ^f^	0.996
0 MPa	0.497 ± 0.011 ^b^	0.053 ± 0.004 ^b^	0.870 ± 0.014 ^e^	0.997
20 MPa	0.234 ±0.008 ^c^	0.023 ± 0.002 ^c^	0.947 ± 0.017 ^d^	0.996
40 MPa	0.218 ± 0.007 ^d^	0.019 ± 0.002 ^d^	0.972 ± 0.015 ^c^	0.997
60 MPa	0.167 ± 0.007 ^e^	0.015 ± 0.001 ^e^	0.980 ± 0.017 ^b^	0.996
80 MPa	0.142 ± 0.005 ^f^	0.013 ± 0.001 ^f^	0.984 ± 0.019 ^a^	0.995
100 MPa	0.125 ± 0.006 ^g^	0.012 ± 0.001 ^f^	0.986 ± 0.017 ^a^	0.996

Different letters in the same column indicate significant difference (*p* < 0.05) between treatments.

## Supplementary Materials

Supplementary File 1
