# Supplementary material for: Effects of High-Pressure Homogenization on the Structural, Physical, and Rheological Properties of Lily Pulp
Source: Foods. 2019 Oct 10;8(10):472. doi: 10.3390/foods8100472 (PMC6835810; doi:10.3390/foods8100472)
Supplement: Supplementary File 1 [file foods-08-00472-s001.pdf]

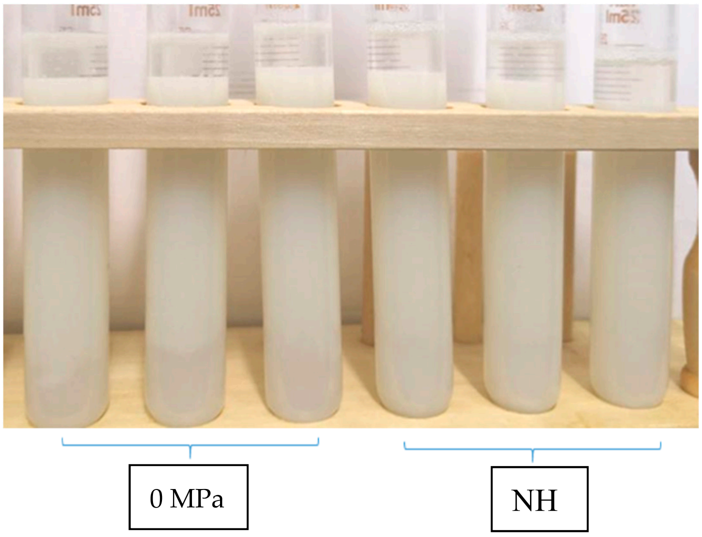

Figure S1 Macroscopic observations of NH (non-homogenized) sample and 0 MPa processed samples after 1 d at 25 °C.

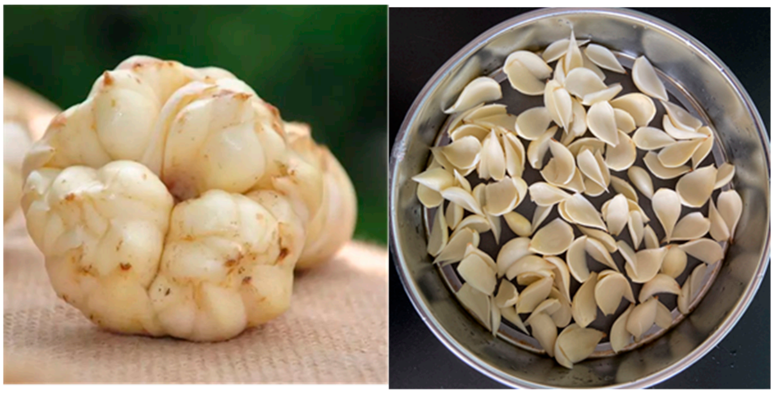

Figure S2. A photo of fresh lily bulbs.
